# Machine learning applied to transcriptomic data to identify genes associated with feed efficiency in pigs

**DOI:** 10.1186/s12711-019-0453-y

**Published:** 2019-03-13

**Authors:** Miriam Piles, Carlos Fernandez-Lozano, María Velasco-Galilea, Olga González-Rodríguez, Juan Pablo Sánchez, David Torrallardona, Maria Ballester, Raquel Quintanilla

**Affiliations:** 1 Animal Breeding and Genetics Program, Institute of Agriculture and Food Research and Technology (IRTA), Torre Marimon s/n, 08140 Caldes de Montbui, Barcelona, Spain; 2 Computer Science Department, University of A Coruña, Campus Elviña s/n, 15071 A Coruña, Spain; 3 Animal Nutrition Program, Institute of Agriculture and Food Research and Technology (IRTA), Mas de Bover, 43120 Constantí, Spain

## Abstract

**Background:**

To date, the molecular mechanisms that underlie residual feed intake (RFI) in pigs are unknown. Results from different genome-wide association studies and gene expression analyses are not always consistent. The aim of this research was to use machine learning to identify genes associated with feed efficiency (FE) using transcriptomic (RNA-Seq) data from pigs that are phenotypically extreme for RFI.

**Methods:**

RFI was computed by considering within-sex regression on mean metabolic body weight, average daily gain, and average backfat gain. RNA-Seq analyses were performed on liver and duodenum tissue from 32 high and 33 low RFI pigs collected at 153 d of age. Machine-learning algorithms were used to predict RFI class based on gene expression levels in liver and duodenum after adjusting for batch effects. Genes were ranked according to their contribution to the classification using the permutation accuracy importance score in an unbiased random forest (RF) algorithm based on conditional inference. Support vector machine, RF, elastic net (ENET) and nearest shrunken centroid algorithms were tested using different subsets of the top rank genes. Nested resampling for hyperparameter tuning was implemented with tenfold cross-validation in the outer and inner loops.

**Results:**

The best classification was obtained with ENET using the expression of 200 genes in liver [area under the receiver operating characteristic curve (AUROC): 0.85; accuracy: 0.78] and 100 genes in duodenum (AUROC: 0.76; accuracy: 0.69). Canonical pathways and candidate genes that were previously reported as associated with FE in several species were identified. The most remarkable pathways and genes identified were NRF2-mediated oxidative stress response and aldosterone signalling in epithelial cells, the *DNAJC6*, *DNAJC1*, *MAPK8*, *PRKD3* genes in duodenum, and melatonin degradation II, PPARα/RXRα activation, and GPCR-mediated nutrient sensing in enteroendocrine cells and *SMOX*, *IL4I1*, *PRKAR2B*, *CLOCK* and *CCK* genes in liver.

**Conclusions:**

ML algorithms and RNA-Seq expression data were found to provide good performance for classifying pigs into high or low RFI groups. Classification was better with gene expression data from liver than from duodenum. Genes associated with FE in liver and duodenum tissue that can be used as predictive biomarkers for this trait were identified.

**Electronic supplementary material:**

The online version of this article (10.1186/s12711-019-0453-y) contains supplementary material, which is available to authorized users.

## Background

Improving feed efficiency (FE) is a priority for most livestock species because it is a key component of profitability, productivity and sustainability for the meat production industry. Genetic selection has proven to be a successful strategy for this purpose in pigs [1, 2] and other livestock species such as cattle [3], poultry [4] or rabbits [5]. The most widely used measures of FE are: feed conversion ratio, residual feed intake (RFI), residual gain, and the more recently proposed residual intake and weight gain [6], which is an index that combines the latter two measurements. Regardless of which FE indicator is used as selection criterion, individual recording of at least feed intake and body weight gain is required, which is expensive and time-consuming. Thus, obtaining reliable and accurate predictive genetic markers for FE is of paramount interest. Molecular mechanisms that underlie RFI in pigs are still unknown. Several genome-wide association studies [7] and gene expression analyses based on microarrays [8–10] or RNA sequencing (RNA-Seq) [11] have been performed, in an attempt to unravel the genetic architecture of this complex trait. However, results from such studies are not always consistent. In this context, the use of alternative methods that are able to provide a good assessment of the generalizability of the results is of great interest. Machine-learning (ML) algorithms applied to a resampling strategy to predict or classify outputs such as RFI class, can be used for this purpose. In recent years, ML algorithms have been used for the analysis of high-throughput deep sequencing data due to their computational efficiency in finding generalizable patterns from high-dimensional data obtained from a small number of samples.

Our aims were to identify candidate genes associated with FE in pigs and to assess the ability of different ML algorithms to predict the RFI class (high or low) of pigs using transcriptomic information from liver and duodenum tissue.

## Methods

### Animals and feeding

Animal material came from an experiment performed within the framework of the ECO-FCE project (“A whole-systems approach to optimise feed efficiency and reduce the ecological footprint of monogastrics”). Twenty-five sows were inseminated with four Hermitage Maxgro boars. Two hundred and forty-four animals (123 entire males and 121 females) were randomly selected from 25 litters from the same batch and raised in the Hermitage sow herd of GePORK. The trial was conducted during a 4-month period (December 2014-March 2015) on the IRTA Pig Experimental Farm (Monells, Spain). Animals entered the trial when they had a body weight (BW) of 25 kg and remained until slaughter at a BW of ~ 105 kg (69 ± 1 and 153 ± 1 days old at the beginning and at the end of the trial, respectively). They were housed in pens of 10 to 12 randomly distributed individuals. Pigs were randomly assigned to one of the following feeding protocols: (1) a conventional two-phase feeding plan, which provided a standard grower diet used until week 6 of the trial followed by a finisher diet until slaughter; (2) a five-phase feeding plan, in which diet specifications changed at ~ 3-week intervals, applied to pens with pigs of heterogeneous BW; (3) a five-phase feeding plan, similar to the former plan but applied to pens with pigs of homogeneous BW; and (4) an eight- to nine-phase precision-feeding plan applied to pens with pigs of homogeneous BW with non-simultaneous dietary changes that were implemented according to the animals’ BW at ~ 2-week intervals. Throughout all these protocols, animals were fed ad libitum by means of electronic feeders placed in each pen (IVO-station feeder; INSENTEC). Feed composition was similar for all treatments at the beginning and at the end of the trial, but in the multi-phase protocols, different diet specifications were implemented according to either the pen’s average BW (T2 and T3) or the pig’s own BW (T4).

Pigs were weighed individually at the start of the trial, then every 3 weeks (weeks 3, 6, 9 and 12), and at the end of the study (week 14 to 15). Back fat thickness was also measured every 3 weeks. Average daily gain (ADG) was computed as the difference in BW at the end and the beginning of the trial divided by time elapsed in days. Daily back fat thickness gain (BFG) was computed as the difference in back fat thickness at the end and the beginning of the trial. Individual feed intake (FI) was recorded by the electronic feeders (IVO-station feeder; INSENTEC). Animal care and procedures were performed according to Spanish and European regulations about the protection of animals used in experimentation, following national and institutional guidelines for the Good Experimental Practices, and approved by the Ethical Committee of the Institute of Agriculture and Food Research and Technology (IRTA).

### Phenotypic analyses of feed efficiency and choice of animals for gene expression analyses

Upper and lower extreme animals for FE were identified using the RFI criterion, which considered within-sex regression coefficients and was computed by the following formula:

$$RFI_{ijk} = FI_{ijk} - \left[ {S_{j} + \beta_{{MBW_{j} }} \times MBW_{i} + \beta_{{ADG_{j} }} \times ADG_{i} + \beta_{{BFG_{j} }} \times BFG_{i} } \right],$$

where $RFI_{ijk}$ is the residual feed intake of individual $i$, of sex *j* (*j* = 1 and 2), and fed the *k* feeding protocol (*k* = 1 to 4); $FI_{ijk}$ is the daily feed intake of individual $i$ during the analysed period; $S_{j}$ is the effect of sex $j$ on FI; $MBW_{i}$, $ADG_{i}$ and $BFG_{i}$ are the metabolic body weight, the ADG, and the BFG of individual $i$, respectively; $\beta_{{MBW_{j} }}$, $\beta_{{ADG_{j} }}$ and $\beta_{{BFG_{j} }}$ are the corresponding partial regressions coefficients (within sex $j$).

At the end of the trial, the five pigs with the lowest, medium, and highest RFI within each combination of sex*feeding strategy were selected; three of these animals were culled because of health problems. After 12 h fasting, the 117 pigs were slaughtered in totally controlled conditions at IRTA’s experimental slaughterhouse at Monells (Girona, Spain), where tissue sampling was performed. For this research, only data from high and low RFI animals were used, and several tissue samples were discarded due to low-quality RNA. Finally, samples from four animals within each sex*treatment*RFI-class combination were retained (five in one case). Thus, the final dataset for the transcriptomic analyses included 65 individuals, 32 high RFI and 33 low RFI pigs from both sexes (16 females and males within each RFI class but for low-RFI males, there were 17 individuals), equally distributed across the four feeding protocols. The four sires were represented in both the high and low RFI groups, with 7, 4, 2, and 7 progeny, respectively, for sires 1, 2, 3, and 4 in the high RFI group and 5, 4, 6, and 6 in the low RFI group.

In order to assess the magnitude of the difference between groups in RFI, a linear model was fitted to the data using the lm() function of R [12]. The initial model included the following factors: FE class (two levels: high and low RFI), feeding protocol (four levels), sex, and the interactions between all these factors. An ANOVA F-test was performed in order to statistically test the significance of the effects of each factor.

### Tissue collection, RNA sequencing and quantification of expression levels

Samples of approximately 1 mg from liver and duodenum were collected after slaughter, immediately submerged in RNA-later and stored at − 80 °C after 24 h. Total mRNA from biological samples was extracted with the RiboPure™ Isolation of High Quality Total RNA kit (Ambion^®^, Austin, TX) following the manufacturer’s recommendations. The isolated mRNA was quantified with a Nanodrop ND-1000 spectrophotometer (NanoDrop products; Wilmington, USA) and checked for purity and integrity with a Bioanalyzer-2100 (Agilent Technologies, Inc.; Santa Clara CA, USA).

Libraries of the 130 RNA samples (65 from duodenum and 65 from liver) were generated using the TruSeq RNA Sample Preparation Kit (Illumina Inc), according to protocols recommended by the manufacturer. Each library was paired-end sequenced (2 × 75 bp), using the TruSeq SBS Kit v3-HS, on an Illumina HiSeq 2000 platform at *Centro Nacional de Análisis Genómico* (CNAG; Barcelona, Spain).

The generated raw reads were quality-checked by FASTQC (Babraham Bioinformatics; http://www.bioinformatics.babraham.ac.uk/projects/fastqc/). Reads were mapped to the reference pig genome Sscrofa 10.2 and the annotation database Ensembl Genes 86 (http://www.ensembl.org/info/data/ftp/index.html) by using STAR v. 2.5.2a [13]. The resulting aligned sequences were merged with Samtools [14] and quantification of the total number of genome-mapped reads to a gene was performed with HTSeq. 0.6.1p2 [15]. Subsequently the RNA-Seq expression data were normalized using the edgeR package [16].

### Analysis of genes associated with feed efficiency by machine learning

Gene expression levels were used as the only information (i.e. predictor variables or features) to classify animals into an RFI class (i.e. animals having either a low or high RFI). The quality of this classification can be considered as a measure of the degree to which RFI differences are explained by differences in gene expression (assuming that the classification algorithm is appropriate). Therefore, we considered that the genes with a more informative expression level for classification for RFI were those that were involved in determining the FE of the animal or, at least, to be highly correlated with them.

Before performing the animal classification by ML, the existence of potential batch effects on gene expression data was tested and adjusted for, and feature selection of the most informative genes was performed. Then, classifications with different ML techniques and features sets were performed using a nested resampling strategy. The methods used in each of these steps are described below.

#### Detection of batch effects

One common issue with RNA-Seq data is the presence of batch effects that can arise, for instance, from laboratory conditions, day of processing, or technician differences. Data normalization does not remove these effects that can affect subsets of genes in a different way. Detection and removal of these batch effects are necessary since they can potentially lead to incorrect conclusions and reduce the accuracy of the analyses. Batch effects have been reported in several published high-throughput studies [17] but few methods have been developed to detect them in high-dimensional expression datasets [18]. Among these, principal component analysis (PCA) is commonly [19] used for data reduction through linear combinations of the dataset that better explain data variation [20]. In this study, a PCA of RNA-Seq expression data was computed and components that correlated with biological or technical variables (e.g. sex, feeding system, and date of pre-processing) were identified. Since the number of genes was large (> 12,000), it is advisable to perform a dimensionality reduction for more stable results with the PCA. Thus, only the 500 most variable genes based on their within-tissue expression level were used in the PCA analysis. Then, RNA-Seq data were adjusted for the first two principal components (PC) using a linear regression analysis.

#### Feature selection

One of the main objectives of our work was to identify the smallest number of genes that yielded the highest possible classification quality. There are three main approaches in ML to perform dimensionality reduction: filter, wrapper, and embedded approaches, for which a good review is in [21]. In this study, the filter approach was chosen because of its computational speed, scalability, and independence from the specific ML technique used for classification [22]. Features were ranked using their importance measure in an unbiased random forest (RF) algorithm based on conditional inference [22]. This nonlinear and non-parametric method can be applied to a wide range of problems, even if they are nonlinear and involve complex interaction effects, and if the number of data is much smaller than the number of predictors. The “cforest” function for conditional inference trees in the “party” R package was used for these analyses. It performs sampling without replacement (instead of bootstrap sampling), which is the only approach that guarantees reliable variable selection and produces unbiased variable importance measures, even in situations in which the predictor variables have different scales of measurement or different numbers of categories [22]. The tuned hyper-parameter for this ML algorithm was the number of input variables that were randomly sampled as candidates at each node, the testing value ranging from 5 to the square root of the number of inputs. The number of trees was fixed to 1000.

Variable importance can be measured in different ways [22]. In this study, we used conditional permutation importance for correlated predictors [23] to rank the genes because of the existence of co-expression patterns among them. In this method, permutation is performed within groups of observations that are defined by the values of the remaining predictor variables; its advantage, compared to univariate screening methods, is that it accounts for the impact of each predictor variable in multivariate interactions with other predictor variables. Machine-learning algorithms were tested with different subsets of data that contained an increasing number (50, 75, 100 and 125) of the most informative genes according to this criterion. As a reference, we also tested the quality of the classification using the expression data of all genes.

#### Machine-learning algorithms for classification

The classification of animals for RFI level (low or high) using gene expression levels as predictor variables was performed using the four ML methods described in the following: support vector machines (SVM), random forest (RF), elastic net (ENET), and prediction analysis for microarrays, also known as nearest shrunken centroids (PAM). These ML algorithms were implemented using the R package “mlr” [24], which is an interface for a large number of classification and regression techniques that allows results from different ML algorithms to be compared under the same conditions and to find the most suitable hyper-parameters for each ML method automatically, ensuring that results are reliable and not biased.

Support vector machine [25–27] is a very well-known ML technique that has been used in several domains with good results in most cases. In fact, SVM and RF are considered state-of-the-art ML algorithms. The main objective of SVM is to find the hyperplane that best separates the samples into high and low RFI, while maximizing the distance between samples and the hyperplane, thus finding the best possible generalization hyperplane [28]. An important element of this algorithm is the kernel and one of the most used kernels is the Gaussian radial basis (RBF) because almost every surface can be obtained with it [29]. One of the main parameters in a SVM is the “cost parameter” (“C”), which is a trade-off between the classification error and the simplicity of the hyperplane decision surface. The other hyper-parameter of SVM regarding the Gaussian function inside the RBF kernel is “gamma”. Performance of SVM is very sensitive to changes in this parameter. Both hyper-parameters were tuned by testing values in powers of two between − 12 and 12. The”e1071” R package was used for the analyses.

Random forest [30] combines several classification trees, which are fitted to subsamples of the original sample-set using randomly selected subsets of predictor variables. From the complete “forest”, a single global prediction is obtained as an average (in case of regression) or majority vote (in case of classification) of the prediction of all trees. The advantages of this method are: (1) it is simple and results are easy to interpret in the case of few predictors; and (2) it can be applied to many problems, even if there are high-order interaction effects or non-linear relationships between the variables. The “randomForest” R package was used for the analysis.

Elastic net [31] is a regression method that combines the penalty approach of ridge regression ($\lambda_{1} \times \left[ {\mathop \sum \nolimits_{j = 1}^{p} \beta_{j}^{2} } \right]$) and the penalty approach of lasso ($\lambda_{2} \times \left[ {\mathop \sum \nolimits_{j = 1}^{p} \left| {\beta_{j} } \right|} \right]$) in a mixture of the two (the elastic net penalty is $\lambda \times \left( {\left( {1 - \alpha } \right) \times \left[ {\mathop \sum \nolimits_{j = 1}^{p} \left| {\beta_{j} } \right|} \right] + \alpha \times \left[ {\mathop \sum \nolimits_{j = 1}^{p} \beta_{j}^{2} } \right]} \right)$, with $\alpha = \frac{{\lambda_{2} }}{{\lambda_{1} + \lambda_{2} }}$ and the $\beta$’s being the regression coefficients of the multiple regression). When the number of predictors is much larger than the number of data ($p > > n$), EL allows more than $n$ predictor variables to be selected out of $p$ candidates. In addition, it can select groups of correlated variables, as in the case of genes sharing the same biological pathway, whereas Lasso tends to select only one variable from a group of correlated variables. The function “cv.glmnet” from the “glmnet” R package was used. The value of $\alpha$ and the coefficient $\lambda$ for the EL penalty were tuned by testing values ranging from 0 (Lasso) to 1 (ridge regression) for $\alpha$ (0, 0.15, 0.25, 0.35, 0.5, 0.65, 0.75, 0.85 and 1), while the optimal $\lambda$ parameter was internally found by cross-validation. Variable importance was estimated as the sum of the regression coefficients of each predictor variable in the different fitted models.

Prediction analysis for microarrays or nearest shrunken centroids [32] is based on the “nearest centroid” method, which computes the standardized centroid for each class and shrinks it towards the overall centroid for all classes by an amount named “threshold”, which is tuned. The predicted class assigned to a new sample is the class for which the centroid is closest in terms of squared distance. A gene that is shrunk to zero for all classes is eliminated from the classification rule and genes with a stable expression within samples of the same class receive a higher weight than genes with a more variable expression. Therefore, this algorithm automatically performs gene selection and can improve classification accuracy by reducing the effect of non-informative genes. The “pamr” package of R was used for the analysis. The hyper-parameters “threshold” (defined above) and “threshold.scale” (a class –dependent scaling factor for the within-class standard deviation) were tuned in order to find the optimal values.

#### Resampling strategy

Nested resampling [33] was performed in order to obtain reliable performance estimates for the learners and to quantify the generalization ability of the generated classifier model. It consists of two nested resampling loops. In the outer resampling loop, a tenfold cross-validation was performed by randomly dividing the dataset in 10 groups of equal size, taking into account the fact that data were slightly unbalanced. Thus, the subsets were stratified in order to keep a similar number of the less frequent class in each subset. One group was used as an outer validation set and the remaining nine groups were used as outer training set. The process was repeated 10 times, with a different group of data used as a validation set each time, resulting in 10 pairs of training/validation sets. Parameter tuning was done for each outer training set by executing the inner resampling loop, which also consisted of a tenfold cross-validation, resulting in one set of selected hyper-parameters for each outer training set. The learner was fitted on each outer training set using the corresponding selected hyper-parameters (from a grid of possible values) and its performance was evaluated on the corresponding outer validation set. The “mlr” [24] package makes it easy to perform all the necessary tasks and to aggregate results obtained from each ML algorithm, comparing them under exactly the same conditions. All analyses were performed using the BioCAI HPC cluster facility at the University of A Coruña.

#### Classification performance measure

Several performance measures are available for a classification problem: the mean misclassification error, accuracy, and measures based on ROC (receiver operating characteristic) analysis. In this work, the area under the ROC (AUROC) was used to guide the search process, since this measure is not conditioned by the number of samples [34] and is independent of the threshold used for classification. Sensitivity (i.e. the proportion of actual positives that are predicted as positive) and specificity (i.e. the proportion of actual negatives that are predicted as negative) were also computed. From the point of view of selection to improve FE in an animal population, specificity would be the most important performance measurement given that low RFI (i.e. more efficient) was considered to be the negative class.

### Functional annotation of the most informative genes

Gene function classification and pathway analyses were performed for the genes that were selected in both tissues as the best predictive set for FE. Before performing the functional analysis, the orthologous human gene names of the pig Ensembl gene ID were retrieved from the Ensembl Genes 89 Database using the Biomart software [35]. Gene ontologies (GO), canonical pathways, and biological functions that were significantly (p_adj_ < 0.05) enriched in the list of genes, each selected as contributor to the classification, were determined using the ClueGO V2.1.7 plug-in of Cytoscape V [36] and the Core Analysis function included in the Ingenuity Pathway Analysis software (IPA; Ingenuity Systems).

## Results

### Phenotypic analysis

**Fig. 1:**
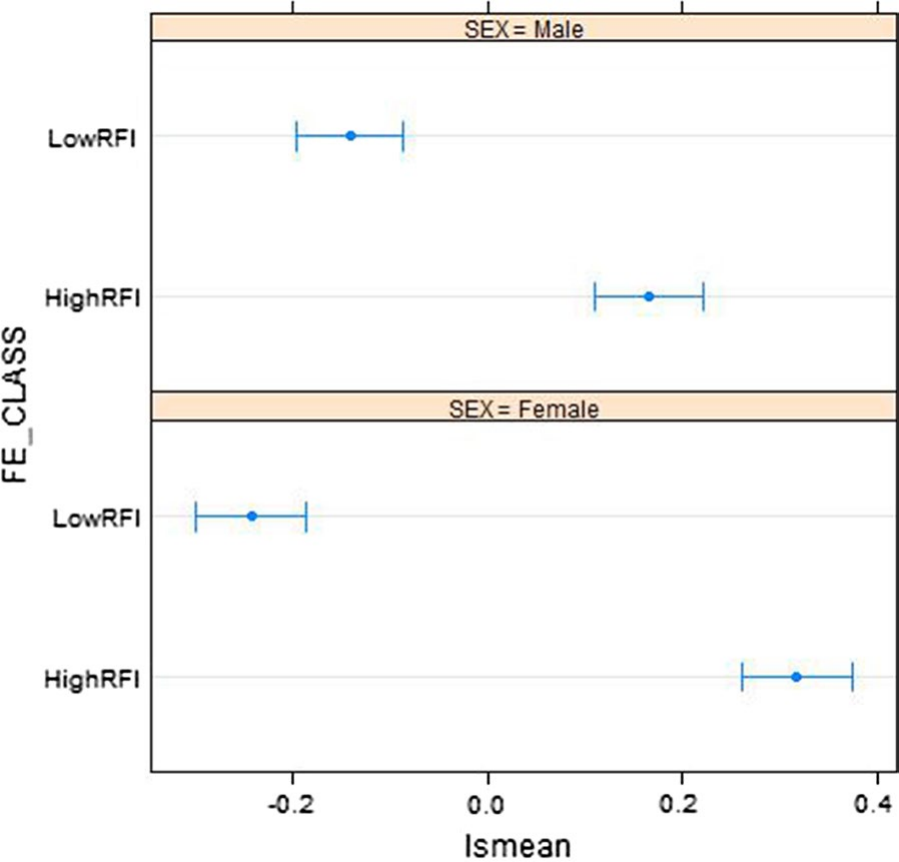
Least square means and confidence intervals for residual feed intake (RFI) by sex and RFI class (low and high)

Raw means for RFI were 0.002 and 0.158 kg for male and female pigs, respectively. Since the feeding strategy had no significant effect on RFI, it was not included in the final model of analysis. Figure 1 shows the 95% confidence intervals for the least square means of RFI for the different levels of FE class and sex. Differences between FE classes were larger in females than males. Differences, in absolute value, between low and high RFI groups were equal to 0.56 ± 0.04 kg (which corresponds to 3.5 standard deviation units) in females and 0.31 ± 0.04 kg (1.9 standard deviation units) in males.

### Identification of genes involved in feed efficiency

**Fig. 2:**
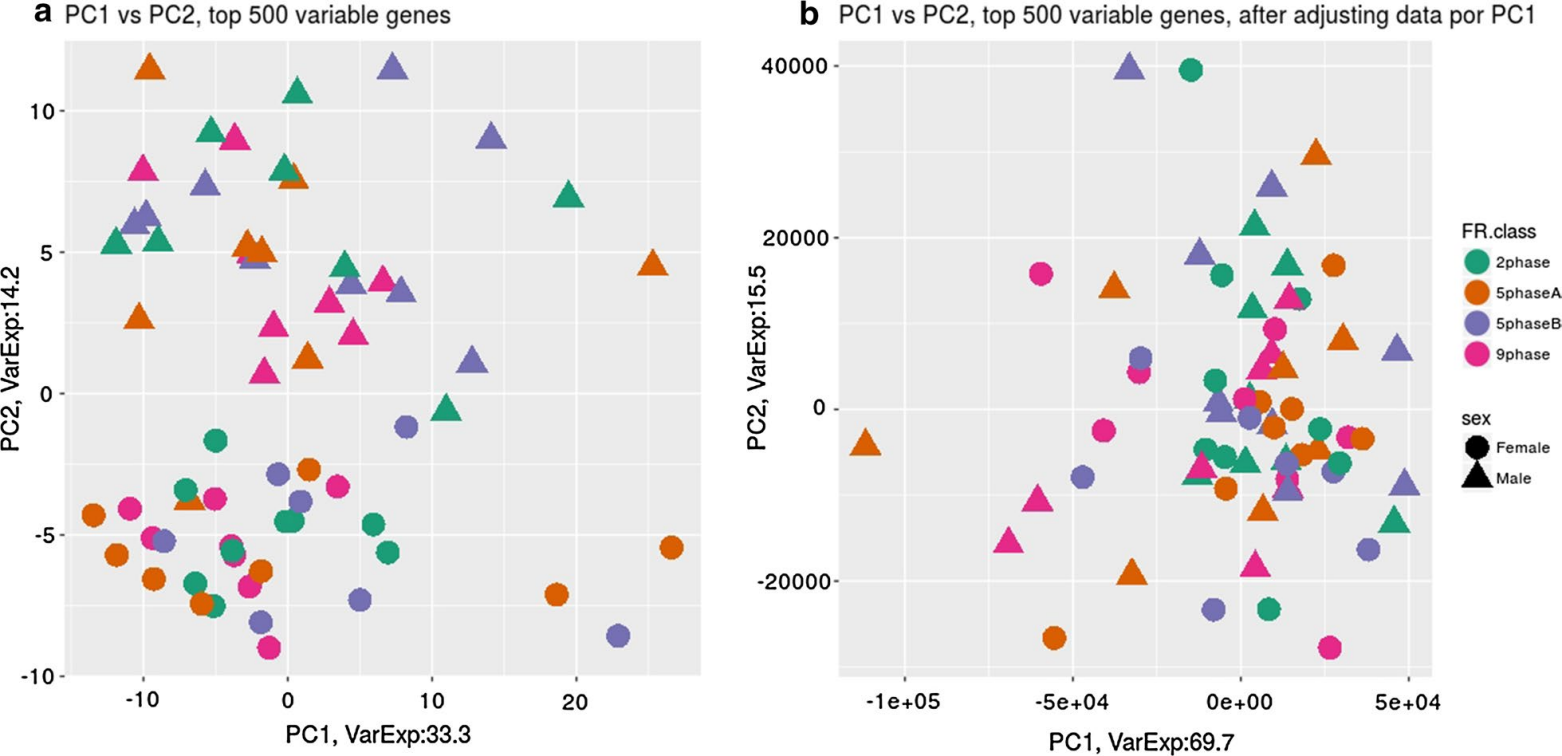
Principal components 1 (PC1) and 2 (PC2) of RNA-seq expression data from liver of animals under different feeding regimes (FR class) and of different sex, before (**a**) and after (**b**) adjusting for PC1. VarExp: percentage of total variance explained by the principal component

**Fig. 3:**
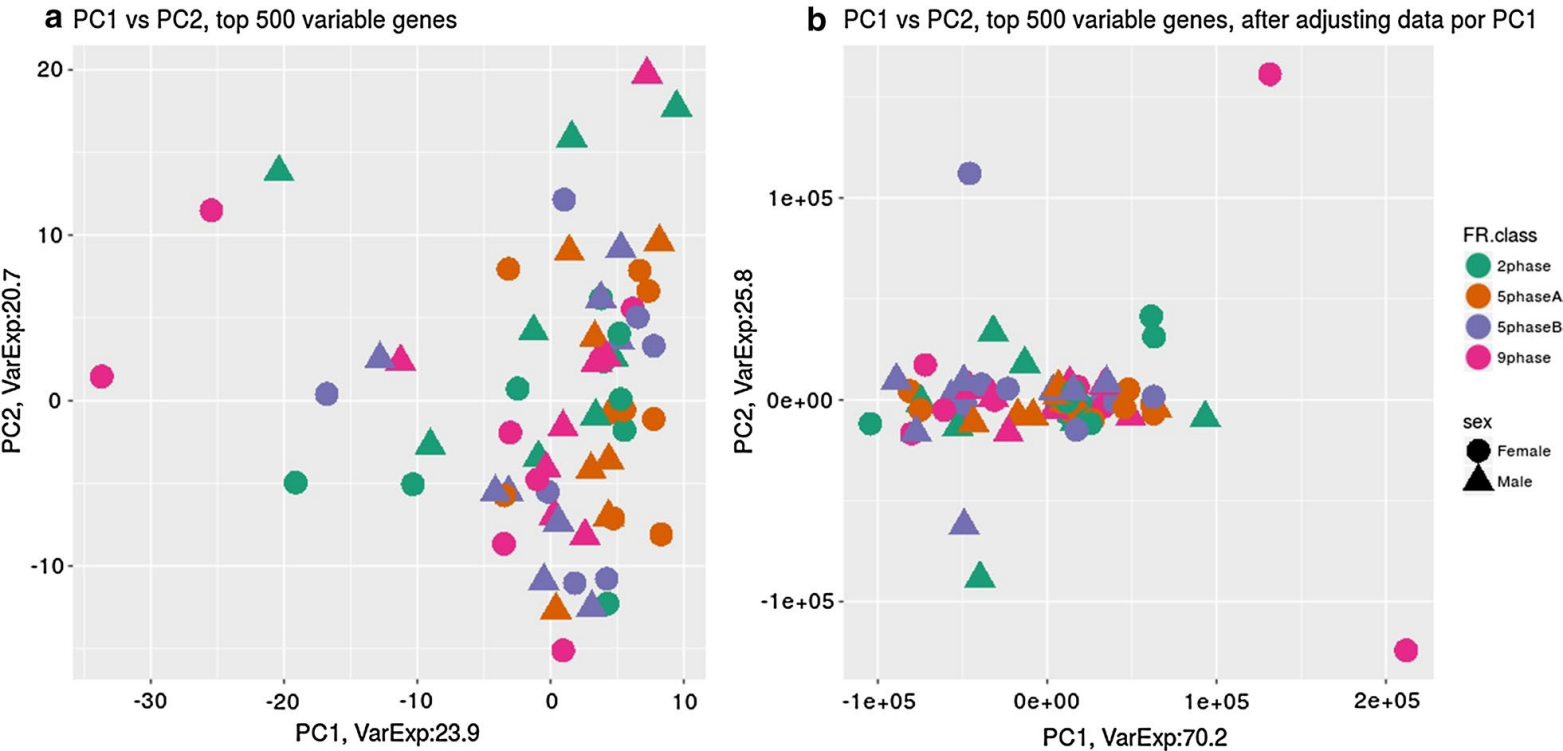
Principal components 1 (PC1) and 2 (PC2) of RNA-seq expression data from duodenum of animals under different feeding regimes (FR class) and of different sex, before (**a**) and after (**b**) adjusting for PC1. VarExp: percentage of total variance explained by the principal component

To assess the presence of batch effects in the RNA-Seq data, we performed a PCA of gene expression data from liver (Fig. 2) and duodenum (Fig. 3) tissue using the 500 most variable genes within each tissue. Whereas no differences between samples associated to any known factor were identified in duodenum (Fig. 3a), a batch effect associated with sex was identified in the liver dataset, for which the second PC clearly separated samples according to this factor (Fig. 2a). The association between sex and expression in liver was removed after adjusting RNA-Seq data by PC1 (Fig. 2b), which explained 33% of the variation, while PC2 only explained 14%. Finally, it is worth noting that gene expression levels did not differ between the feeding protocols and that the interactions of feeding protocol with other factors in the model were not significant. Thus, these interactions were ignored in subsequent analyses of the RNA-Seq data.

In a first step of the classification process, genes were ranked according to their importance measure using an unbiased RF algorithm based on conditional inference [22]. Then, four subsets with increasing numbers (50, 75, 100 and 125) of the most informative genes were obtained for both the liver and the duodenum gene expression datasets. Those subsets of RNA-Seq data, with or without adjustment for batch effects, were used as predictors of the RFI class in a second step. The classification performance of four ML techniques was measured and compared in terms of the AUROC of 10 test sets from the tenfold outer cross-validation analysis with the best model parameters for each dataset and ML algorithm. Results corresponding to liver and duodenum datasets are presented separately below.

#### Classification of pigs for RFI based on liver gene expression data

**Fig. 4:**
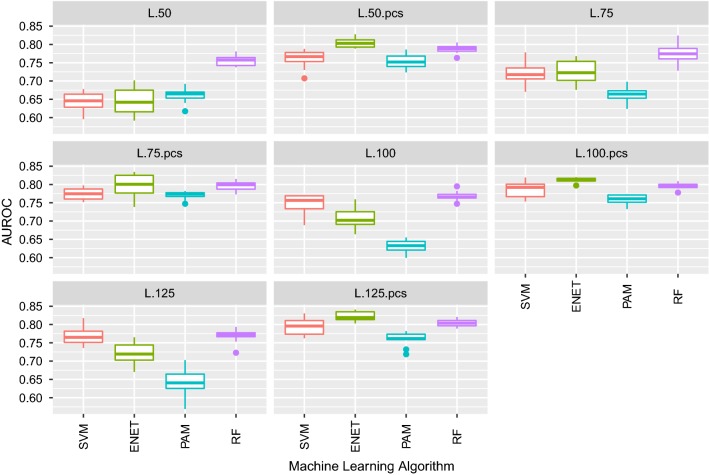
Boxplot of AUROC of the classification of pigs on RFI in 10 test sets (from tenfold cross-validations). Classification was based on liver RNA-Seq expression data corresponding to different subsets of genes (50, 75, 100 and 125), either raw or pre-corrected by batch effects (suffix “pcs”), and was performed using support vector machine (SVM), elastic net (ENET), nearest shrunken centroids (PAM) and random forest (RF) algorithms

We observed clear improvement in classification performance of all ML algorithms when classification was based on PC1 and liver gene expression data that were pre-corrected for PC1 compared to the performance obtained with the original datasets. In addition, the stability of the results tended to increase when using pre-corrected data, as shown in the lower interquartile range for the AUROC results (Fig. 4). This indicates that the bias due to batch effects was removed with this correction. No further improvement in the classification performance was obtained by adjusting for PC2 or for both PC (results not shown).

The ML algorithm that led to the best performance for all pre-corrected datasets was ENET (cross-validated lambda). The “one-standard-error” rule for selecting the best model was used, as recommended by [33] and previously proposed by [37]. The best classification was obtained with 125 genes, which corresponded to a mean AUROC value of 0.79 when the positive class was high RFI (Fig. 4). The highest stability of results, measured in terms of dispersion of AUROC, was observed when only the expression levels of the 100 most informative genes were used as predictors (interquartile interval = 0.008), followed by using 125 genes (interquartile interval = 0.021).

**Fig. 5:**
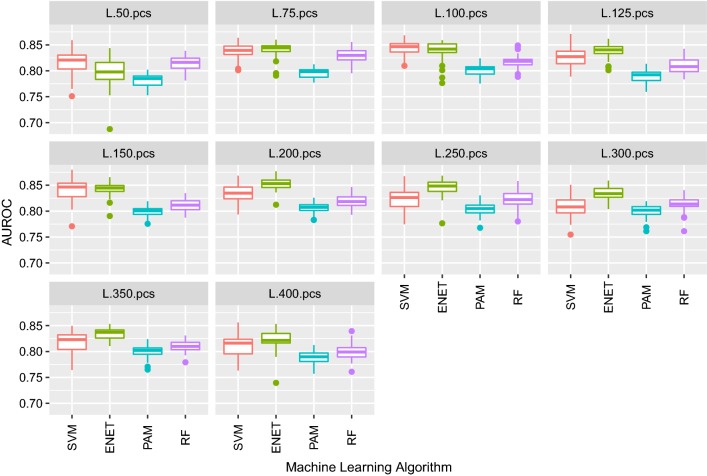
Boxplot of AUROC of the classification of pigs on RFI in 10 test sets (from tenfold cross-validations). Classification was based on liver RNA-Seq expression data corresponding to different subsets of genes (50, 75, 100, 125, 150, 200, 250, 300, 350 and 400), pre-corrected by batch effects, and was performed using support vector machine (SVM), elastic net (ENET), nearest shrunken centroids (PAM) and random forest (RF) algorithms

Since the best performance was obtained with the subset that had the largest number of genes (i.e. 125 genes), we performed a new classification task increasing the number of genes in the subsets up to 400 genes after adjustment for PC1 (i.e. 50, 75, 100, 125, 150, 200, 250, 300, 350 and 400 genes in each subset). In this new analysis, all processes were repeated 5 times to better assess the stability of the results. In terms of AUROC (Fig. 5), the SVM and ENET algorithms performed similarly when the number of genes was less than 200 (Fig. 5), but ENET had the best classification when the number of genes was 200 or more, while the PAM algorithm performed the worst in all cases. ENET also showed a greater stability of results in most cases, since the interquartile range was smaller. Combining accuracy [see Additional file 1: Figure S1] and stability, the best performance was obtained with ENET using the subset with the 200 most important genes as predictors.

A further increase in the number of genes used for classification did not improve its quality in any case, on the contrary it led to a decrease in AUROC (Fig. 5) and accuracy [see Additional file 2: Figure S2]. The performance of RF and PAM algorithms was less affected by non-informative genes than ENET or SVM, being SVM the most affected algorithm. Thus, when the number of genes increased from 200 to 400, the mean AUROC decreased by 0.03 points of the maximum for both SVM and ENET (Fig. 5). As a reference, classification based on all sequenced genes (i.e. 12,560 genes) after correction for batch effects using the ENET algorithm resulted in mean values for AUROC, accuracy, sensitivity, and specificity of 0.46, 0.50, 0.90, and 0.09, respectively, indicating no ability to classify pigs for RFI based on gene expression in liver samples when using all genes.

Elastic net performs an internal selection of the predictor variables (i.e. candidate genes). See Additional file 3: Table S1 lists the genes selected, the differential expression between the two groups of animals for each gene, the number of times that a particular gene was selected (votes), and the cumulated value of the coefficients of the linear regression equations as a measure of the importance of each gene across all classification processes (“sumbetas”). Since a tenfold cross-validation was repeated 50 times in the outer loop, the maximum number of times that a particular gene could be selected (number of “votes”) across all classification processes is 500 (5 × 10 × 10). Each of the 200 selected genes was selected 500 times, which means that they all contributed to the classification. Figure 7a shows the importance of the 40 top contributing genes to the classification of pigs into the high or low RFI class based on gene expression in liver samples.

#### Classification of pigs for RFI based on duodenum gene expression data

**Fig. 6:**
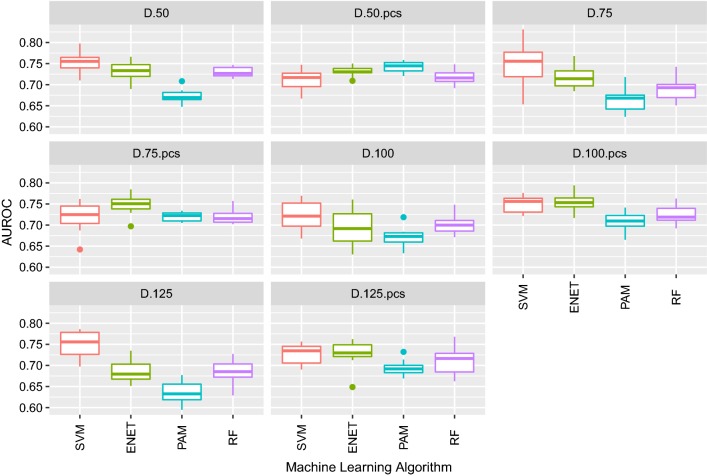
Boxplot of AUROC of the classification of pigs on RFI in 10 test sets (from tenfold cross validations). Classification was based on duodenum RNA-Seq expression data corresponding to different subsets of genes (50, 75, 100 and 125), either raw or pre-corrected by batch effects (suffix “pcs”), and was performed using support vector machine (SVM), elastic net (ENET), nearest shrunken centroids (PAM) and random forest (RF) algorithms

Figure 6 and see Additional file 4: Figure S3 show the results from the classification of pigs for RFI based on duodenum RNA-Seq expression data with the four ML algorithms by applying the best model parameters for each algorithm. When duodenum RNA-Seq expression data were used, correction of the data by PC slightly improved the quality of the classification in most of the analyses regardless of the method and subset of genes used. However, correction for batch effects had less impact on the quality of the classification than for the liver gene expression data (Fig. 6). For example, when the duodenum RNA-Seq data was corrected by PC1, the mean AUROC of the classification with ENET increased by only 0.05 to 0.06, depending on the number of genes in the prediction dataset.

In general, the quality of the classification with the duodenum dataset was worse than that obtained with the liver dataset. The best classification in terms of AUROC was obtained when the ENET algorithm was used with the expression data from the 100 most informative genes after correction for PC (mean AUROC = 0.76); accuracy, specificity, and sensitivity of this classification were 0.69, 0.66, and 0.71, respectively [see Additional file 4: Figures S3]. Increasing the number of genes used for classification did not lead to an improvement in AUROC for any ML algorithm.

**Fig. 7:**
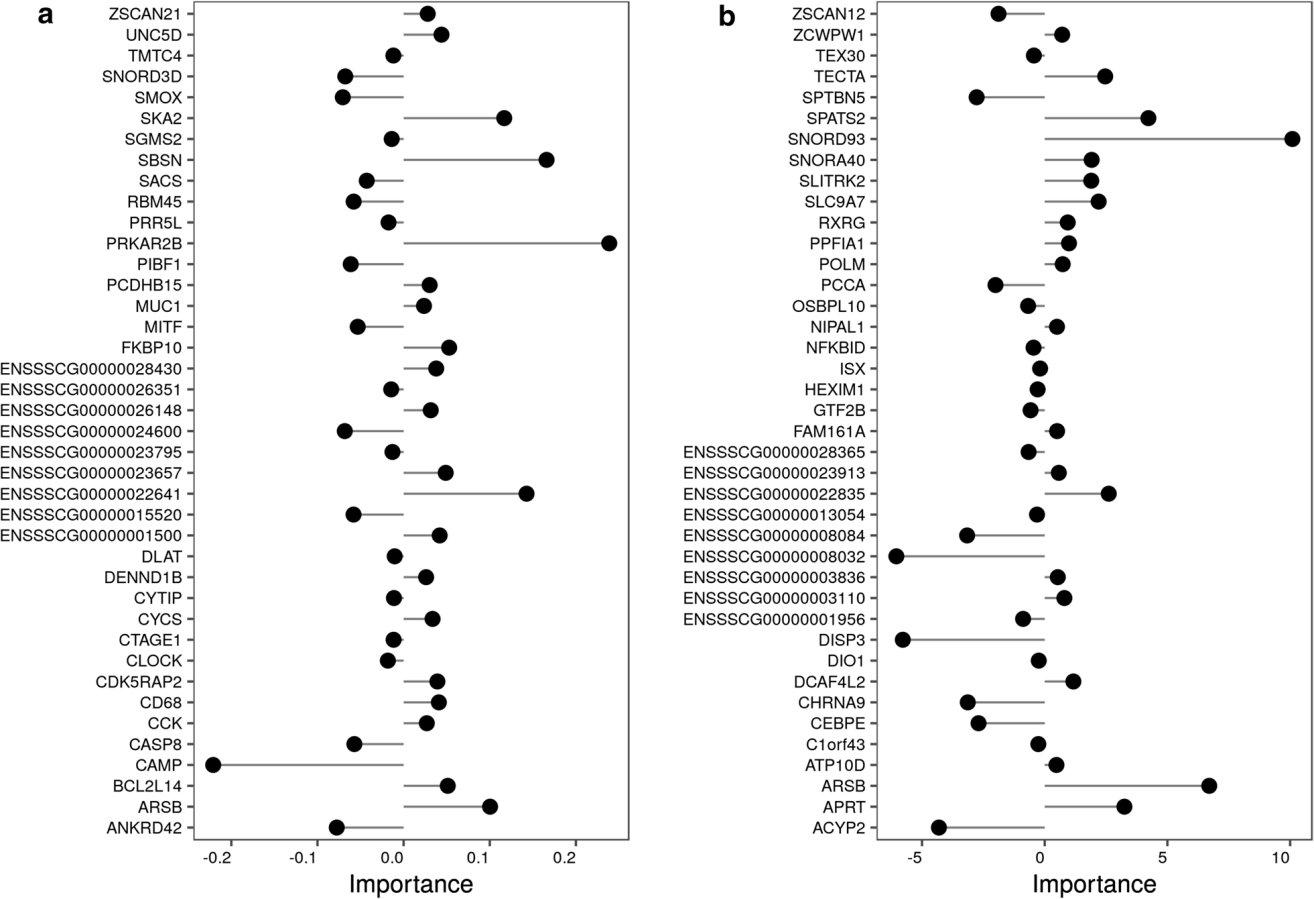
Importance of the 40 top genes contributing to the classification of samples into the high or low RFI class. **a** Liver. **b** Duodenum

In the case of duodenum, each of the 100 selected genes was selected as contributor to the classification task across all the classification processes. See Additional file 5: Table S2 shows the genes, the differential expression between the high and low RFI groups of animals for each gene, the number of times they were selected in the validation process (“votes”), and the importance measure defined above (“sumbetas”). Figure 7b shows the importance of the 40 top contributing genes to the classification of pigs into the high or low RFI class based on the duodenum gene expression data.

### Functional analysis of the most informative genes for RFI classification

We carried out a functional characterization of the genes for which the expression level allowed the best classification of pigs regarding RFI. Of the genes selected for each tissue based on their contribution to the classification, only those that had an orthologous human gene were used in this functional analysis, resulting in 180 and 86 candidate genes [see Additional file 6: Table S3] that were submitted to IPA for the liver and duodenum data analyses, respectively.

The most significant networks identified by IPA are listed in Table 1. For liver, the second most represented network was associated with carbohydrate metabolism, cellular growth and proliferation, and organismal development functions, while networks associated with cellular development, cellular assembly, and cellular organization were found for both tissues [see Additional file 7: Figure S4].

**Table 1:** Top networks enriched by the most informative genes for RFI classification in liver and duodenum tissues

Top networks				
Tissue	ID	Associated network functions	Score	Molecules
Liver	1	Cancer, dermatological diseases and conditions, organismal injury and abnormalities	48	25
	2	Carbohydrate metabolism, cellular growth and proliferation, organismal development	45	24
	3	Cell Morphology, cellular assembly and organization, cellular development	43	23
	4	Cellular development, hematological system development and function, lymphoid tissue structure and development	31	18
	5	Cancer, hematological disease, immunological disease	24	15
Duodenum	1	Cell death and survival, connective tissue development and function, skeletal and muscular system development and function	42	18
	2	Cell-to-cell signaling and interaction, inflammatory response, cellular assembly and organization	33	15
	3	Cell death and survival, cellular compromise, cancer	33	15

For liver, the following canonical pathways were found to be significantly enriched in genes that were selected in liver as the best predictive set for FE: melatonin degradation II, tryptophan degradation X (mammalian, via tryptamine), sirtuin signaling pathway, PPARα/RXRα activation, GPCR-mediated nutrient sensing in enteroendocrine cells, and xenobiotic metabolism signalling, among others [see Additional file 8: Table S4]. Among others, the *CCK*, *CLOCK*, *IL4I1*, *PRKAR2B*, and *SMOX* genes are involved in these overrepresented pathways and they were among the top genes that contributed to the classification (Fig. 7a). The *SLC2A10* and *SLC5A10* genes, which are related with carbohydrate transmembrane transporter activity, and the *PFKM* and *HOOK1* genes, which are related with the glycolytic process, were also in the list of selected genes for liver tissue.

In duodenum, among the identified canonical pathways, it is worth mentioning: NRF2-mediated oxidative stress response, role of pattern recognition receptors in the recognition of bacteria and viruses, aldosterone signalling in epithelial cells, production of nitric oxide and reactive oxygen species in macrophages, cholecystokinin/gastrin-mediated signalling, unfolded protein response, protein ubiquitination pathway, and IL-8 signalling among others [see Additional file 8: Table S4]. Some of the genes of these canonical pathways were *CASP1*, *DNAJC6*, *DNAJC1*, *MAPK8* and *PRKD3*.

## Discussion

Feed efficiency is one of the most economically important traits in animal production and its improvement is an on-going topic of research in different areas such as nutrition and genetics. Genetic selection on FE was demonstrated to be efficient in several experiments on pigs and thus is a key strategy to improve FE [1, 2]. However, its implementation is difficult due to the high cost and difficulty to obtain phenotypic records for this trait. Thus, finding reliable and accurate predictive genetic markers that are associated with FE has particular relevance for genetic selection, since they would help increase selection accuracy and provide predictions of FE early in life, thus reducing the generation interval in selected lines.

The goodness of a classification procedure relies on achieving a good generalization performance (i.e. high accuracy of the classification or prediction in a testing subset of data) and uniqueness (i.e. stability) of the results. In spite of the demonstrated good performance of ML techniques in different areas of research such as industry or medicine, few applications of these methods have been reported in animal and plant breeding. Non-parametric methods, such as some of those used in our study, allow making predictions of complex traits, such as FE, without imposing a specific structure to the association between trait phenotype and predictor variables (e.g. expression levels, genotypes). This is especially relevant for the study of traits that have a complex genetic architecture [38].

In this study, we evaluated gene expression levels between male and female pigs with high or low extreme RFI. The difference in RFI between high and low groups of animals was clear and was larger in females than in males. In a study based exclusively on records from females, Ramayo-Caldas et al. [39] found differences between high and low RFI groups of animals for other measures of FE such as feed conversion ratio and residual intake and weight gain. Analysis of these alternative FE traits in the complete dataset (results not shown) led to results that were consistent with those of Ramayo-Caldas et al. [39] in females.

In our study, animals were exposed to different feeding protocols, thus an interaction between genotype and feeding protocol was expected. However, our results did not show any significant effect of the feeding regimen and/or its interaction with other factors of the model for FE or gene expression data. Since all animals were slaughtered under the same conditions, we expect that expression profiles may reflect differences between high and low RFI groups.

Sex had an important effect on gene expression levels in liver but not in duodenum tissue. Previous studies have reported sexual dimorphism at the gene expression level in relevant metabolic tissues such as liver, muscle, and/or adipose tissue in pigs [40–42], cows [43], humans, rat, and mouse [44, 45]. Sexually dimorphic genes have also been observed in the small intestine of rat and mouse species [46, 47]. Van Nas et al. [45] observed a sex-specific regulation that was affected by gonadal hormones at the hepatic gene expression level, which included metabolism of steroid hormones and drugs and control of fatty acid homeostasis among the principal cellular functions affected by sex [41, 44, 45].

In pigs, the liver regulates the whole-body energetic homeostasis involved in different lipid metabolism functions, whereas absorption of food takes place in the small bowel. Our results indicate a sex effect on gene expression in the liver but not in the duodenum, and this may be related with the specific biological functions that the 500 most variable genes in liver and duodenum tissues are involved in. Accordingly, the overrepresented biological processes and KEGG pathways among the 500 most variable genes included steroid metabolic process, drug and xenobiotics metabolism, and oxidative phosphorylation and lipid-related biological functions in the liver, and generation of precursor metabolites and energy, fat digestion, absorption of lipids and minerals, and immune response-related functions in the duodenum.

Differential expression analysis was performed in several consecutive steps. In a first step, RNA-Seq data were adjusted by potential group effects due to sex, differences in environmental conditions at different dates of sample processing, or some other unnoticed factors, which could affect the RNA-Seq data and lead to incorrect conclusions if they were correlated with the outcome of interest [17]. While the effect of sex was strongly correlated with PC2, which explained 14% of the variation in RNA-Seq data in liver, no other known potential factor of variation (e.g. feeding plan) was identified. In general, after correction of RNA-Seq data by PC1 (which explained 33 and 24% of the variance in expression levels in liver and duodenum, respectively), classification quality was clearly improved for most of the ML methods and subsets of genes, especially in the liver dataset. However, no additional improvement was obtained by including a correction for PC2, which suggests that the effect of un-modelled factors could be more important than the effects of sex on the gene expression data.

Some studies have shown the importance of feature selection methods [21] for selecting informative genes prior to classification [47]. Feature selection methods remove irrelevant and redundant features to improve the classification accuracy. In a second step of our analyses, the predictor variable importance was assessed by using an unbiased RF algorithm based on conditional inference [22]. Its advantage over univariate screening methods is that it takes interactions between predictor variables into account. This method was demonstrated to produce unbiased variable importance measures even when the predictor variables have different scales of measurement or different numbers of categories [23]. “Conditional permutation importance” was used as the variable importance measure because of the high correlation between predictors. After ranking gene expression variables according to this criterion, four ML algorithms (SVM, ENET, PAM and RF) were evaluated for their performance to classify extreme animals on RFI using gene datasets that included an increasing number of the most informative genes. The evaluated algorithms were chosen because of their proven good performance for classification in many studies.

Our findings demonstrate the negative effect of non-informative genes on the classification, and the need for feature selection. Using RNA-Seq data of all 12,560 genes from liver did not enable ML algorithms to classify extreme animals on RFI (mean AUROC = 0.45 and mean accuracy = 0.50 with ENET). In contrast, performance was quite good when information for classification was limited to the 200 most informative genes (mean AUROC = 0.85 and mean accuracy = 0.78 with ENET). With the RF and PAM algorithms, the performance decreased slightly when more, less or non-informative predictors, were included due to the built-in feature selection. In spite of the regularization, the parametrically structured model ENET was slightly more affected by those predictors than the RF and PAM algorithms, maybe because of the excess of parameters added to the model that could lead to overfitting. The SVM algorithm was most affected by including more, less or non-informative predictor variables. Similar results have been reported by Kuhn and Johnson [48].

Stability of the performance of a classifier, as measured by dispersion parameters of the performance measures, reflects the reliability and uniqueness of the results. In 50 runs of tenfold cross-validation analysis, the interquartile intervals of AUROC ranged from 0.01 to 0.02 across methods when 200 genes were used and from 0.01 to 0.03 otherwise. This indicates a good stability of the results regardless of the method and subset of genes used.

We have found a list of genes in liver and duodenum that allow the classification of extreme animals on RFI. Remarkably, several of these genes are components of canonical pathways that were previously associated with FE traits in different species [39, 49–52], which highlights the relevance of these genes and pathways in the genetic determination of FE. For example, in the duodenum dataset, it is worth mentioning the *DNAJC6*, *DNAJC1*, *MAPK8* and *PRKD3* genes, which are involved in pathways such as the NRF2-mediated oxidative stress response and the aldosterone signaling in epithelial cells. These pathways have been identified in several transcriptome analyses that were carried out on animals with divergent FE phenotypes. In spite of differences depending on the tissue and species analyzed, it is generally admitted that the more efficient pigs have a lower production of reactive oxygen species (ROS) and less oxidative stress [39, 50–53]. In chickens, the NRF2-mediated oxidative stress response was higher in high FE individuals [49]. In a previous study of our group [39] in which a different approach (weighted gene co-expression analysis; WGCNA) was used to identify functionally-related modules of co-expressed genes associated with FE traits, genes encoding heat shock proteins and DNAJ were also identified. In addition, heat shock genes were also found to be associated with variation in weight gain and feed intake in beef steers [50, 51]. DNAJC6 and DNAJC1 are components of the conserved DNAJ/HSP40 family of proteins, which play a central role in protein homeostasis and are essential for normal growth and development [54, 55]. The *MAPK8* and *PRKD3* genes encode serine/threonine-protein kinases of the MAP kinase and protein kinase D families, respectively, which are involved in a wide variety of cellular processes. Recently, the *MAPK8* gene, which is part of the IL-8 signalling canonical pathway [56], was shown to be differentially expressed in the muscle transcriptome of pigs that had divergent feed efficiencies and product qualities. In our study, both the *MAPK8* and *PRKD3* genes were also identified as components of the IL-8 signalling canonical pathway. Another pathway in which these genes are involved is the cholecystokinin/gastrin-mediated signalling pathway, which is linked with the GPCR-mediated nutrient sensing in enteroendocrine cells in liver.

Among the canonical pathways that were most significantly enriched by the set of informative genes in liver, we identified melatonin degradation II. Different studies have associated the melatonin degradation pathway with FE traits in beef cattle [50, 57]. Melatonin is a hormone that plays a major role in the regulation of metabolic processes, internal circadian temporal organization, and control of body weight [58]. In rats that were exposed to continuous light, a decrease in melatonin levels was associated with lower food intake, but with an increase in FE and visceral adiposity compared to animals exposed to continuous dark or to a 12-h/12-h light/dark cycle [59]. The *SMOX* and *IL4I1* genes were identified as components of this pathway. In addition, the *CLOCK* gene was identified as a relevant gene in the liver as a component of the sirtuin signalling and PPARα/RXRα activation pathways. The protein encoded by *CLOCK* is a transcriptional activator that regulates circadian rhythms. It should be noted that one of the chronobiological actions of melatonin is the circadian synchronization of hepatic metabolism [58], which indicates a tight relationship among these overrepresented pathways. The *CCK* and *PRKAR2B* genes are components of the GPCR-mediated nutrient sensing in enteroendocrine cells. The protein encoded by *PRKAR2B* is one of the regulatory subunits of the protein kinase A (PKA) and is involved in regulation of energy balance and adiposity [60]. CCK is a neuropeptide and a gut hormone that acts as a satiety signal for regulating food intake [61]; the *CCK* gene was recently shown to be associated with FE in laying ducks [62]. In addition, in combination with *MKKS*, *CCK* has been associated with response to food and regulation of appetite GO biological processes.

Finally, some canonical pathways associated with FE that overlapped between liver and duodenum (*i.e.* aldosterone signalling in epithelial cells and protein ubiquitination pathway) were also identified, although the genes that were identified to be associated with FE differed between liver and duodenum. In a recent study, Ramayo-Caldas et al. [39] also identified an overlap of functions but not of genes associated with FE traits between tissues, which suggests tissue-specific regulation of genes in liver and duodenum tissues or lack of power to identify specific genes.

## Conclusions

Our findings allow us to conclude that the use of RNA-Seq expression data has predictive ability for classifying pigs into high or low RFI groups. Moreover, we demonstrate the good performance of ML techniques for making predictions on complex traits such as FE, in this case based on transcriptomic information. Among the ML algorithms tested, ENET performed the best in all analyses with different tissue transcriptome data and sets of genes used for prediction. Gene expression data from liver resulted in better classification of animals on RFI than expression data from duodenum, and reached a performance of 0.85 in terms of AUROC. We also identified several candidate genes associated with FE, which could be used as predictive biomarkers for this FE. Among the identified most predictive genes were those involved in melatonin degradation II, PPARα/RXRα activation, and GPCR-mediated nutrient sensing in enteroendocrine cells pathways (*SMOX*, *IL4I1*, *PRKAR2B*, *CLOCK* and *CCK* genes in liver), and NRF2-mediated oxidative stress response and aldosterone signalling in epithelial cells (*DNAJC6*, *DNAJC1*, *MAPK8*, *PRKD3* genes in duodenum).

## Additional files

**Additional file 1: Figure S1.** Boxplot of accuracy of the classification of pigs regarding RFI in 10 test sets (from tenfold cross validations). Classification was based on liver RNA-Seq expression data corresponding to different subsets of genes (50, 75, 100 and 125), either raw or pre-corrected by batch effects (suffix “pcs”), and was performed using support vector machine (SVM), elastic net (ENET), nearest shrunken centroids (PAM) and random forest (RF) algorithms.

**Additional file 2: Figure S2.** Boxplot of accuracy of the classification of pigs regarding RFI in 500 test sets (from 50 runs of tenfold cross validations). Classification was based on liver RNA-Seq expression data, pre-corrected for batch effects, corresponding to different subsets of genes (50, 75, 100, 125, 150, 200, 250, 300, 350 and 400) and was performed using support vector machine (SVM), elastic net (ENET), nearest shrunken centroids (PAM) and random forest (RF) algorithms.

**Additional file 3: Table S1.** Genes selected in liver, the number of times that a particular gene was selected (votes) and the cumulated value of the coefficients of the linear regression equations across all the analyses (“sumbetas”).

**Additional file 4: Figure S3.** Boxplot of accuracy of the classification of pigs regarding RFI in 10 test sets (from tenfold cross-validations). Classification was based on duodenum RNA-Seq expression data, either raw or pre-corrected by batch effects (suffix “pcs”), corresponding to different subsets of genes (50, 75, 100 and 125) and was performed using support vector machine (SVM), elastic net (ENET), nearest shrunken centroids (PAM) and random forest (RF) algorithms.

**Additional file 5: Table S2.** Genes selected in duodenum, the number of times that a particular gene was selected (votes) and the cumulated value of the coefficients of the linear regression equations across all the analyses (“sumbetas”).

**Additional file 6: Table S3.** List of most informative genes for RFI classification in liver and duodenum tissues having an orthologous human gene.

**Additional file 7: Figure S4.** Plots of the biological networks most significantly enriched by the most informative genes for RFI classification in liver (A and B) and duodenum tissues (C and D): (A) Carbohydrate metabolism, cellular growth and proliferation, organismal development; (B) Cell morphology, cellular assembly and organization, cellular development; (C) Cell death and survival, connective tissue development and function, skeletal and muscular system development and function; (D) Cell-to-cell signaling and interaction, inflammatory response, cellular assembly and organization. The shape of nodes indicates the functional classes of the gene products.

**Additional file 8: Table S4.** List of canonical pathways identified by IPA from the most informative genes for RFI classification in liver and duodenum tissues.
