# Supplementary figures and images for: Machine learning applied to transcriptomic data to identify genes associated with feed efficiency in pigs
Source: Genet Sel Evol. 2019 Mar 13;51:10. doi: 10.1186/s12711-019-0453-y (PMC6417084; doi:10.1186/s12711-019-0453-y)

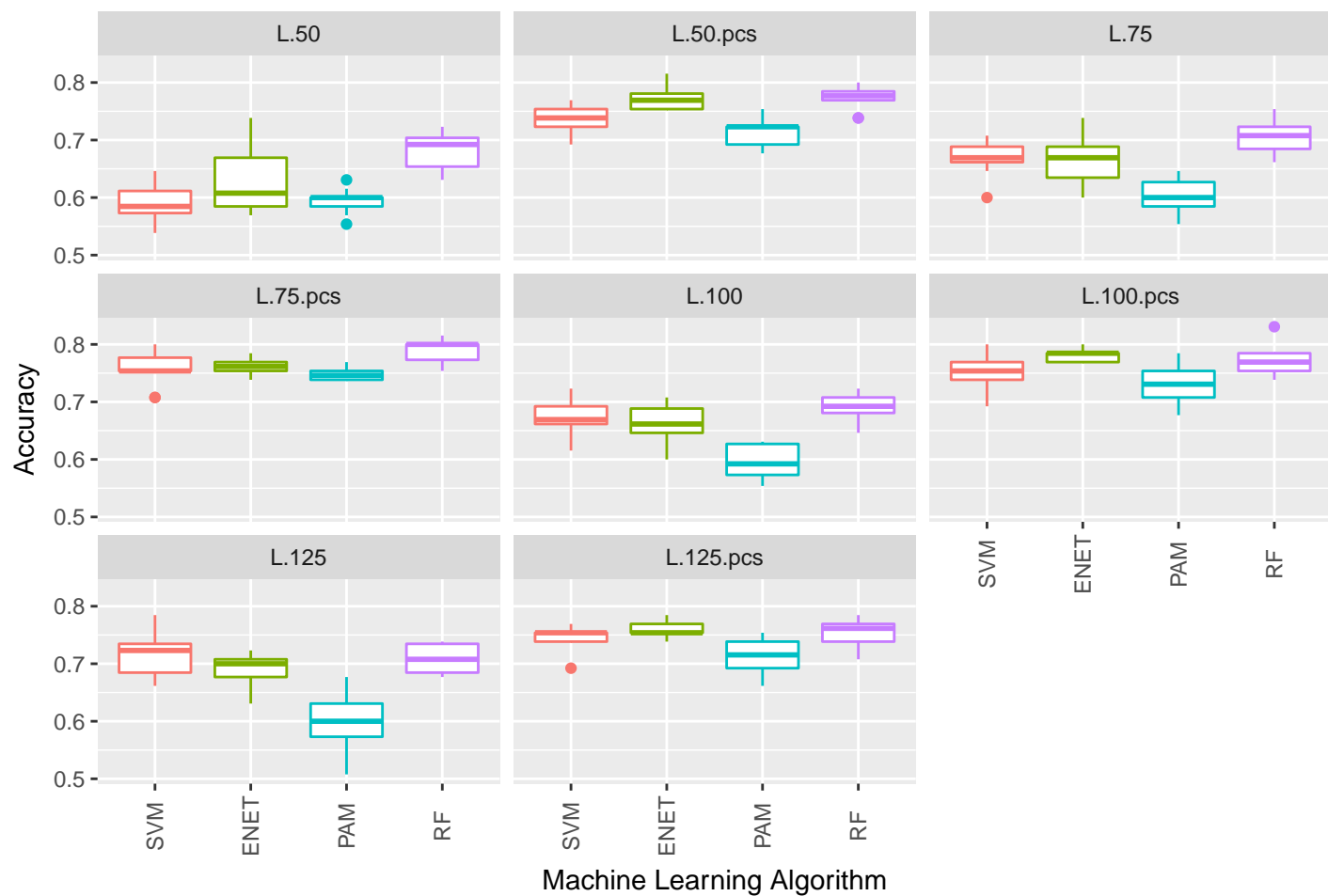

Supplement: Supplementary file 1 — Additional file 1: Figure S1. Boxplot of accuracy of the classification of pigs regarding RFI in 10 test sets (from tenfold cross validations). Classification was based on liver RNA-Seq expression data corresponding to different subsets of genes (50, 75, 100 and 125), either raw or pre-corrected by batch effects (suffix “pcs”), and was performed using support vector machine (SVM), elastic net (ENET), nearest shrunken centroids (PAM) and random forest (RF) algorithms. [file 12711_2019_453_MOESM1_ESM.pdf]

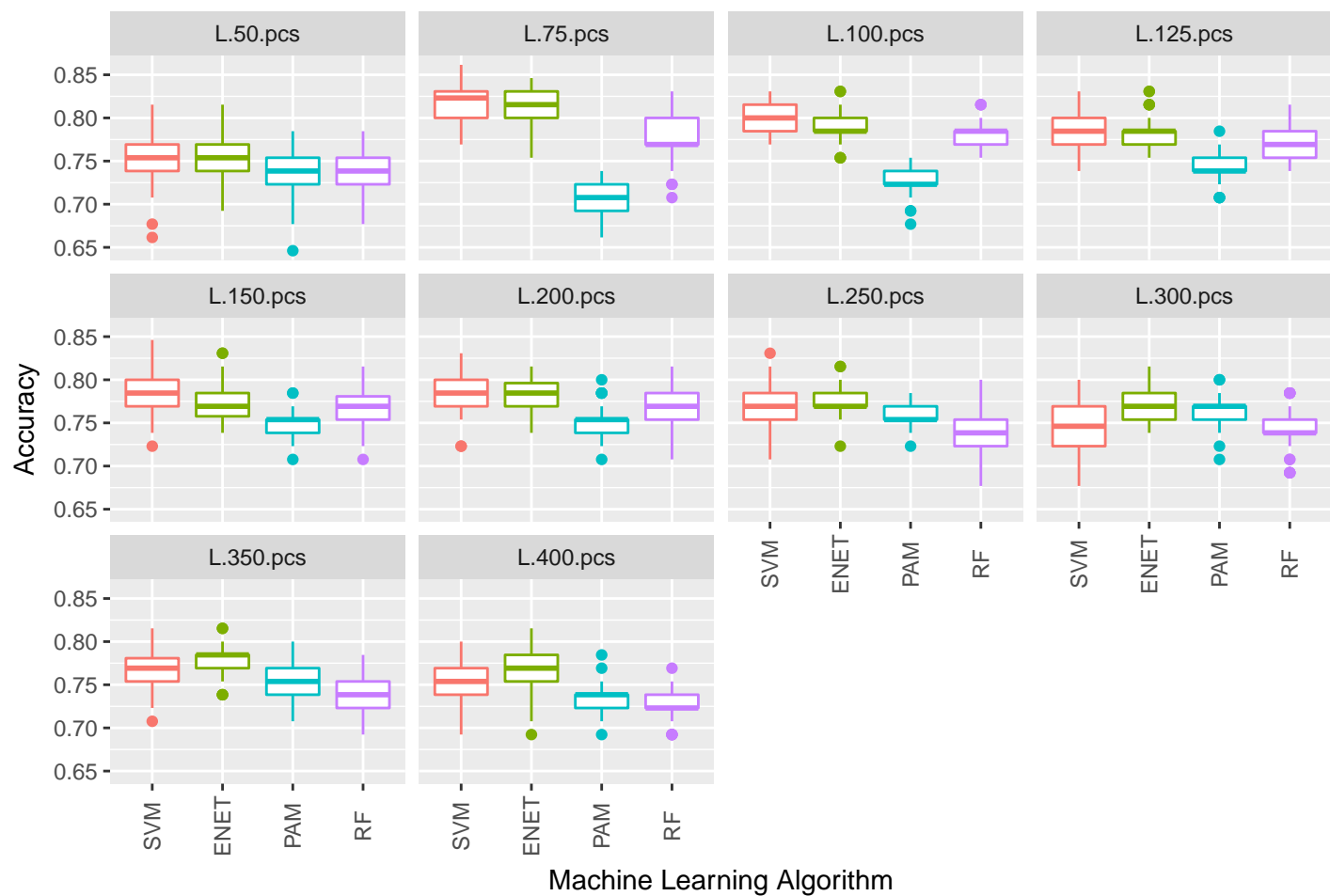

Supplement: Supplementary file 2 — Additional file 2: Figure S2. Boxplot of accuracy of the classification of pigs regarding RFI in 500 test sets (from 50 runs of tenfold cross validations). Classification was based on liver RNA-Seq expression data, pre-corrected for batch effects, corresponding to different subsets of genes (50, 75, 100, 125, 150, 200, 250, 300, 350 and 400) and was performed using support vector machine (SVM), elastic net (ENET), nearest shrunken centroids (PAM) and random forest (RF) algorithms. [file 12711_2019_453_MOESM2_ESM.pdf]

Accuracy

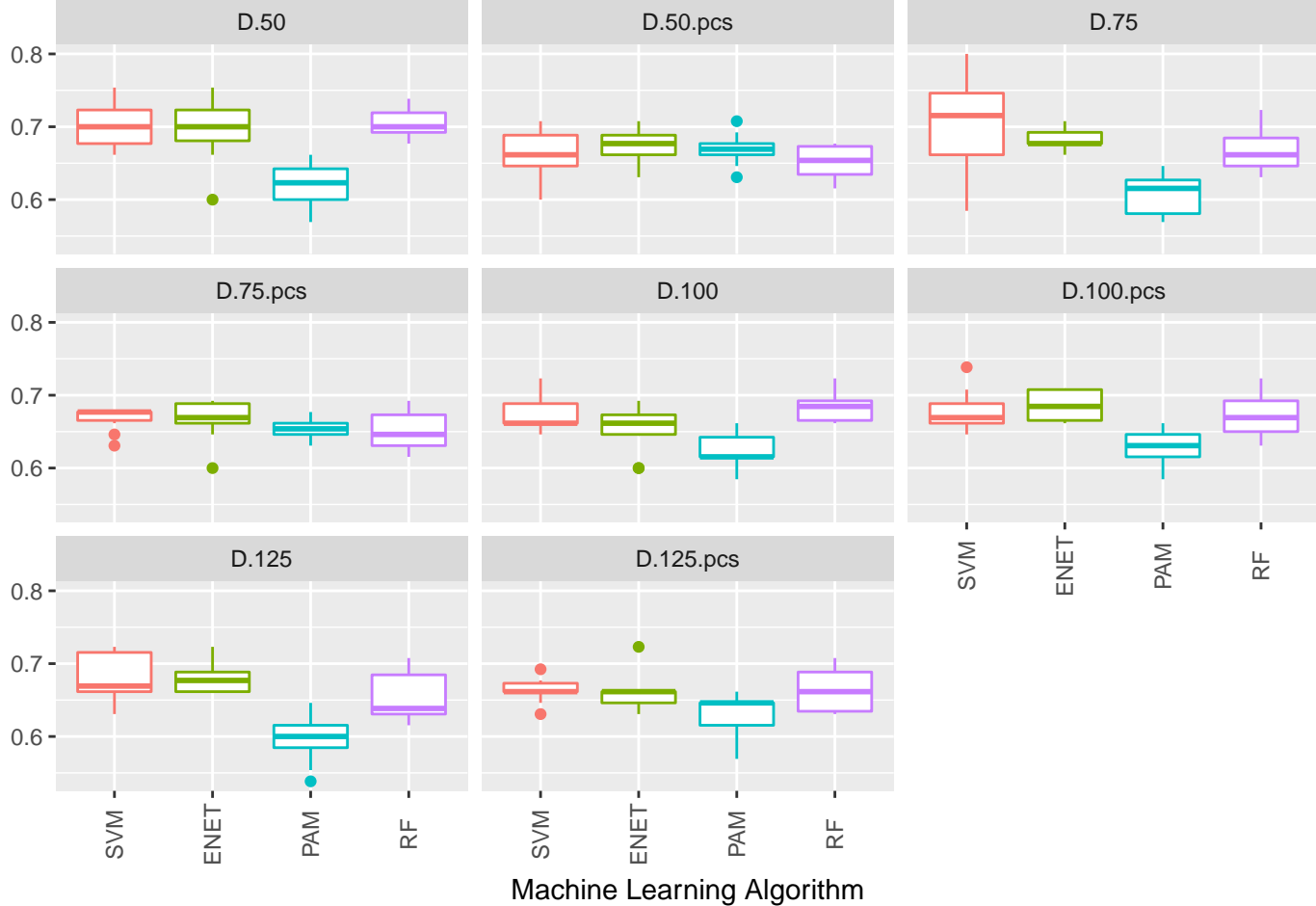

Supplement: Supplementary file 4 — Additional file 4: Figure S3. Boxplot of accuracy of the classification of pigs regarding RFI in 10 test sets (from tenfold cross-validations). Classification was based on duodenum RNA-Seq expression data, either raw or pre-corrected by batch effects (suffix “pcs”), corresponding to different subsets of genes (50, 75, 100 and 125) and was performed using support vector machine (SVM), elastic net (ENET), nearest shrunken centroids (PAM) and random forest (RF) algorithms. [file 12711_2019_453_MOESM4_ESM.pdf]
