# Supplementary material for: Machine learning applied to transcriptomic data to identify genes associated with feed efficiency in pigs
Source: Genet Sel Evol. 2019 Mar 13;51:10. doi: 10.1186/s12711-019-0453-y (PMC6417084; doi:10.1186/s12711-019-0453-y)

Figure S4. Plots of the biological networks most significantly enriched by the most informative genes for RFI classification in liver (A and B) and duodenum tissues (C and D): (A) Carbohydrate Metabolism, Cellular Growth and Proliferation, Organismal Development; (B) Cell Morphology, Cellular Assembly and Organization, Cellular Development; (C) Cell Death and Survival, Connective Tissue Development and Function, Skeletal and Muscular System Development and Function; (D) Cell-To-Cell Signaling and Interaction, Inflammatory Response, Cellular Assembly and Organization. The node color indicates the degree of expression: (red) up-regulated and (green) down-regulated in the Low RFI group relative to the High RFI group. The shape of nodes indicates the functional classes of the gene products.

A


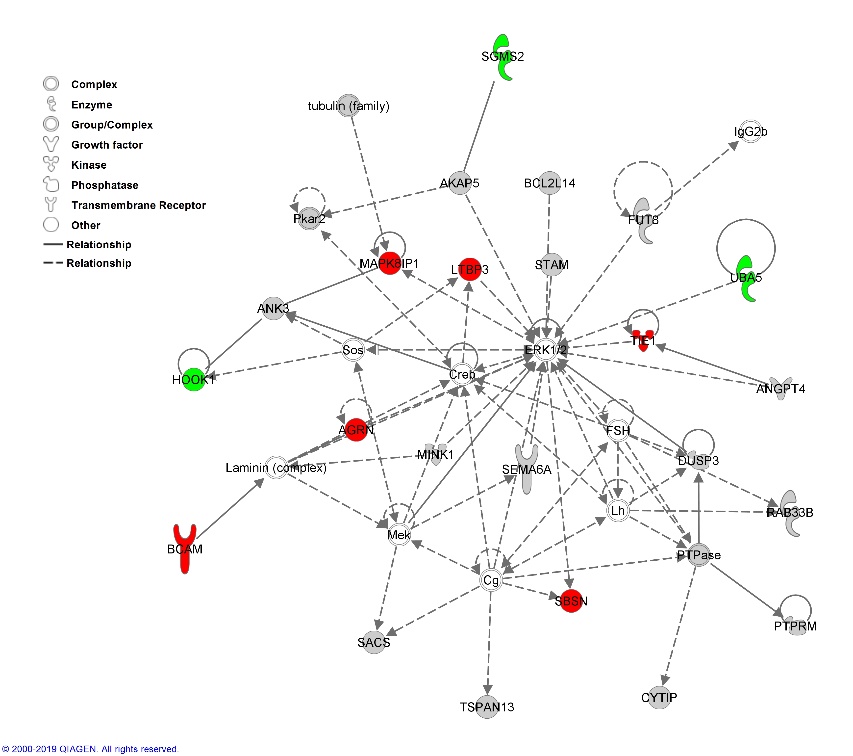


B


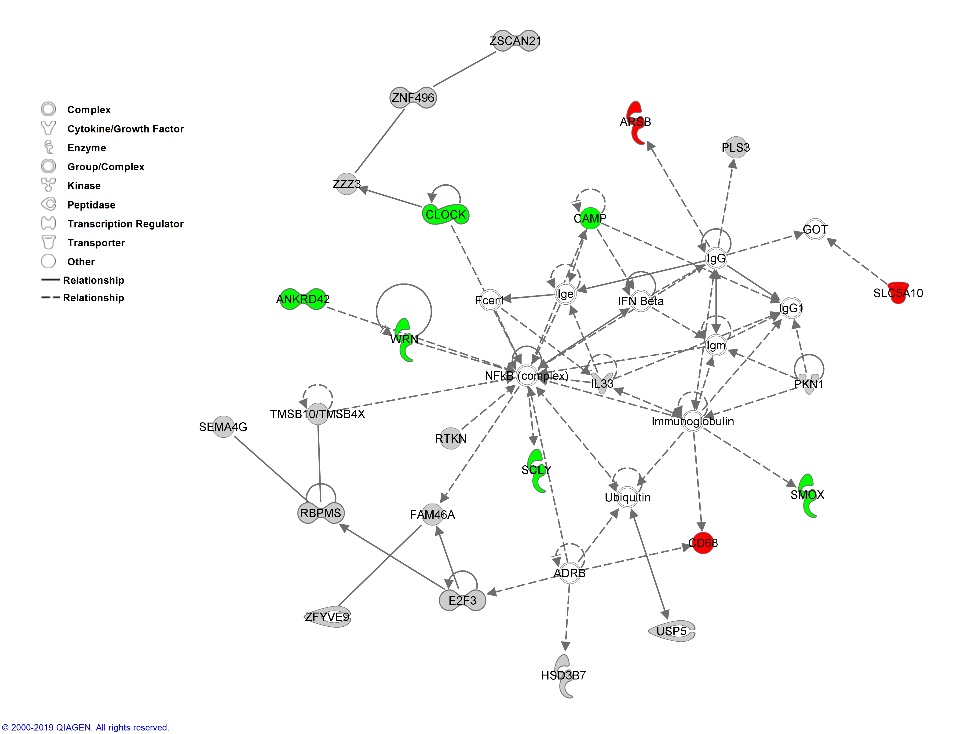


C


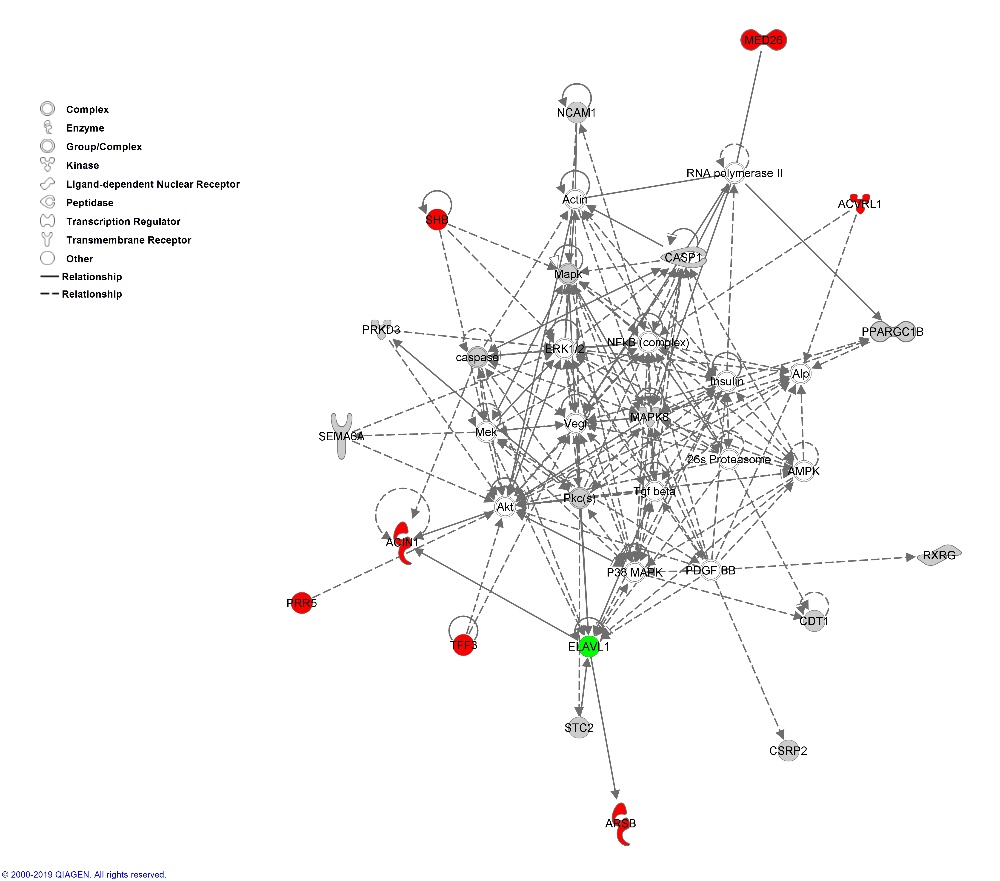


D


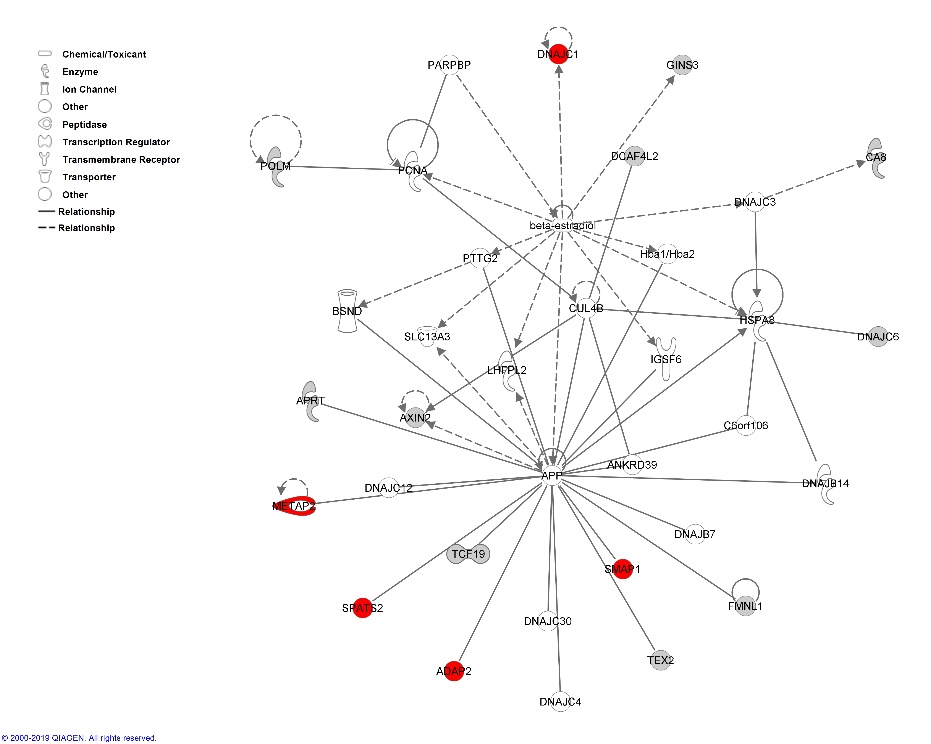

Supplement: Supplementary file 7 — Additional file 7: Figure S4. Plots of the biological networks most significantly enriched by the most informative genes for RFI classification in liver (A and B) and duodenum tissues (C and D): (A) Carbohydrate metabolism, cellular growth and proliferation, organismal development; (B) Cell morphology, cellular assembly and organization, cellular development; (C) Cell death and survival, connective tissue development and function, skeletal and muscular system development and function; (D) Cell-to-cell signaling and interaction, inflammatory response, cellular assembly and organization. The shape of nodes indicates the functional classes of the gene products. [file 12711_2019_453_MOESM7_ESM.docx]
